# Anophthalmic Sockets: A Retrospective Review of Enucleations, Eviscerations, and Exenterations Performed and Managed in a Tertiary Care Hospital

**DOI:** 10.3390/jcm14217764

**Published:** 2025-11-01

**Authors:** Dayna Yong Wei Wei, Jason Timothy Pan, Stephanie Young Ming, Gangadhara Sundar

**Affiliations:** 1 Department of Ophthalmology, National University Hospital, Singapore 119074, Singapore; 2 Yong Loo Lin School of Medicine, National University of Singapore, Singapore 119077, Singapore; 3 Eagle Eye Centre, Singapore 574623, Singapore

**Keywords:** anophthalmia, enucleation, anophthalmic socket, socket management

## Abstract

**Background/Objectives**: Enucleation, evisceration, and exenteration remain essential in ophthalmic practice, despite advances in medical and surgical care. Optimal outcomes rely on meticulous technique, implant selection, and long-term socket rehabilitation. This study reports a single surgeon’s 15-year experience managing anophthalmic sockets at a tertiary referral hospital in Southeast Asia. **Methods**: A retrospective review was conducted. Clinical records were examined for demographics, indications, type of surgery, implant characteristics, wrapping material, complications, and secondary interventions. **Results**: A total of 175 anophthalmic sockets were managed (82 primary, 88 secondary). Mean age was 34.1 ± 30.0 years, 54.9% males. The most common indications were ocular/orbital tumors and trauma. Among primary cases, 70 underwent enucleation, 7 evisceration, and 5 exenteration. The most common implant placed was porous polyethylene (Medpor). Donor sclera was the most frequently used wrapping material. Fifteen (18.3%) primary sockets developed postoperative complications, implant exposure being the most common. Among secondarily managed sockets, more than half had severe complications, particularly severe socket contraction and post-enucleation socket syndrome. **Conclusions**: Anophthalmic sockets remain a complex and challenging condition in ophthalmic practice. Tumors and trauma were the leading indications for globe removal in this cohort. Despite refinements in surgical technique, complications persist, emphasizing the need for multidisciplinary, long-term care to optimize functional and aesthetic outcomes.

## 1. Introduction

An anophthalmic socket refers to an orbit lacking an eyeball but retaining orbital soft tissues and eyelid structures [1]. It is usually unilateral and rarely bilateral. The condition may be developmental, typically recognized at birth from optic vesicle malformation, or acquired. Acquired anophthalmia usually results from surgical removal of the globe for ocular or systemic diseases, advanced intraocular tumors, or trauma [2,3]. Well-managed anophthalmic sockets may not lead to aesthetic or psychological dysfunction [4]. However, when inadequately managed, patients frequently experience medical and functional complications, as well as aesthetic, psychosocial, and economic disruption [5,6]. Rarely, residual malignant or infectious disease may spread intracranially or systemically, posing life-threatening risks [7].

Globe removal is performed by enucleation, evisceration, or, less commonly, exenteration. In enucleation, the entire eyeball is removed, whilst evisceration involves the removal of all intraocular contents while preserving the scleral shell, extraocular muscles, and adnexa [8]. The use of artificial eyes dates back to ancient civilizations: Egyptians and Sumerians fashioned them to decorate mummies and statues [9]. Around 500 BC, Romans used clay eye models to cover phthisical eyes [10]. Eye extirpation was first described by George Bartisch in 1583, though it was previously performed by Johannes Lange in 1555 [11]. Early surgeries were crude and painful, leaving the sockets unsuitable for ocular prosthesis fitting [12]. The procedure was refined in 1841 when O’Ferrall and Bonnet reported their technique of extraocular muscle disinsertion [13]. By the late 19th century, the principles of conjunctival closure and the use of a conformer were adopted [14].

Evisceration, introduced by Bear in 1817 to manage an expulsive hemorrhage [15], was applied by Noyes in 1874 for intraocular infection. Modern modifications employ anterior and posterior sclerotomy techniques to expand the scleral shell size for larger implants (20–22 mm), minimizing the volume deficiency common with smaller implants (13–16 mm) [16].

Orbital exenteration, a radical procedure involving partial or complete removal of the orbital contents and at times the eyelids, was also described by Bartisch in 1583 [17]. The first report of modern total exenteration, however, was published by Golovine in the early 20th century [18]. Recent modifications, such as eyelid-sparing techniques [19], conjunctiva retention [20], and periorbita preservation, aid in facial rehabilitation [21].

With medical and surgical advances, the need for globe removal has reduced significantly. Nonetheless, enucleation, evisceration, and, infrequently, exenteration remain part of ophthalmic practice. Over the years, Oculoplastic surgeons and Ocularists have developed complementary roles in achieving optimal cosmetic and functional outcomes, enhancing patients’ self-esteem and quality of life [22]. We hereby report a single surgeon’s 15-year experience managing anophthalmic sockets at a tertiary referral center in Southeast Asia.

## 2. Materials and Methods

This was a retrospective review of all patients with anophthalmic sockets at our center over a 15-year period (2006–2021). Sockets primarily managed included those that had undergone enucleation, evisceration, or exenteration surgery at the center. Sockets that were secondarily managed included those referred from other centers in Singapore and the region, some of which had undergone prior surgical intervention.

The same surgeon reviewed and operated on all patients. Details, including demographics, underlying disease entity, indication for globe removal, surgical procedure performed, implant type, shape, and size, wrapping material, postoperative rehabilitation, complications, and outcomes were reviewed. Patients with insufficient documentation were excluded. Institutional Review Board approval was obtained prior to data collection and the study conformed to the Declaration of Helsinki.

## 3. Results

A total of 175 anophthalmic sockets were managed over a 15-year period (2006–2021). Of these, 82 orbits were managed primarily with enucleation, evisceration, or exenteration during this period, while 88 were managed secondarily upon referral from elsewhere in Singapore and the region. Five sockets were excluded due to incomplete clinical records. The most common predisposing conditions for both primary procedures and secondarily managed sockets were ocular/orbital tumors (50.0% and 35.2%, respectively) and trauma (17.1% and 35.2%, respectively) (Table 1). These were also the most common indications for enucleation (71.4% of those primary sockets, and 80.0% of those secondarily managed), as well as those referred for secondary socket management post-evisceration (66.7%). All seven primary eviscerations were performed for fulminating infections.

### Primary Sockets

**Figure 1:**
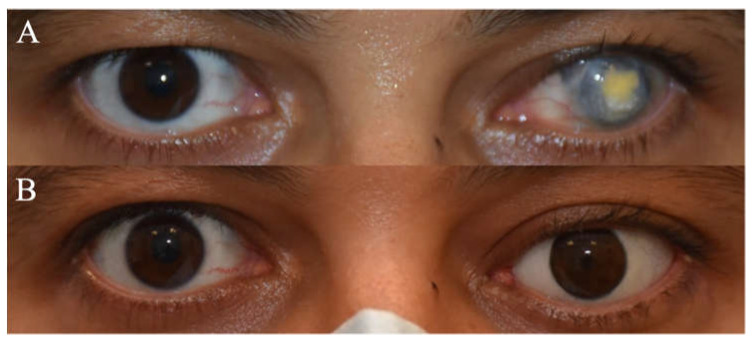
(**A**) Clinical picture of a patient with left eye penetrating trauma who underwent multiple failed corneal grafts. (**B**) Clinical picture post left eye enucleation with a customized prosthesis.

Of the 82 primary sockets, 70 underwent enucleation, 7 underwent evisceration and 5 underwent exenteration (Table 2). All enucleated cases received primary implants, with 1 delayed (Figure 1). Similarly, for sockets that underwent evisceration, all had primary implants, 2 of which were delayed. Of the 5 exenterated sockets, 2 had primary free flap reconstruction and 3 were healed by epithelization through secondary intention.

Of the 77 sockets that underwent enucleation or evisceration, 45 (58.4%) received porous implants: Polyethylene implant (Medpor; Stryker, Kalamazoo, MI, USA)) in 44 sockets and hydroxyapatite implant in 1 socket. The remaining 30 (40.0%) had non-porous implants consisting of 29 acrylic (polymethylmethacrylate) and 1 silicone implant. Information on implant material was not available for 2 (2.6%) sockets. The average implant diameter was 20.5 mm (range: 18–23 mm). Implant shapes are summarized in Table 3; three sockets had an implant that was indeterminate. Of the 70 who had enucleation, 59 (84.3%) used wrapping material. Eleven sockets (15.7%) had myoconjunctivalization. A breakdown of the implant material is reflected in Table 3.

**Figure 2:**
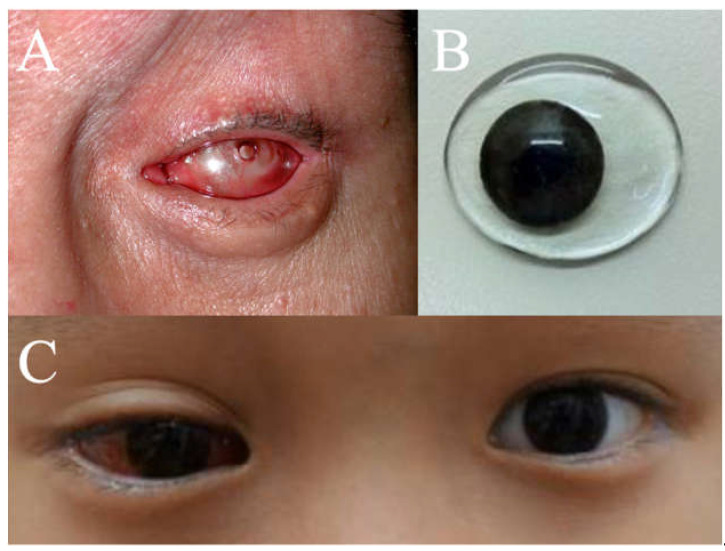
(**A**) Photo post-enucleation with a conformer. (**B**) Iris-painted conformer. (**C**) Iris-painted conformer in a patient at postoperative week 1.

**Figure 3:**
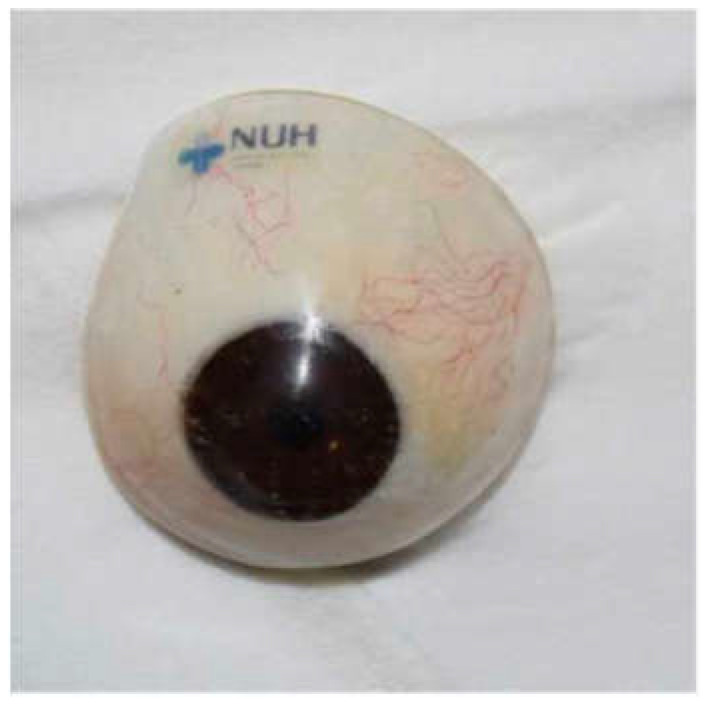
Customized prosthesis.

Postoperatively, all 77 sockets, post-enucleation or evisceration, had conformers placed (Figure 2): 39 stock and 38 iris-painted. After 6–8 weeks postoperatively, 57 patients were fitted with customized prostheses (Figure 3), 13 continued to use stock conformers, and 7 continued to use iris-painted conformers.

**Figure 4:**
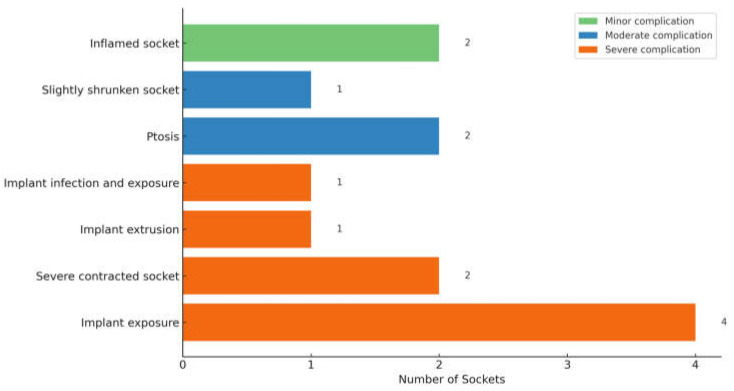
Postoperative Complications observed after Primary Enucleation (n = 13).

None of the 82 primarily managed anophthalmic sockets had intraoperative complications. Fifteen sockets (18.3%) developed postoperative complications: the evisceration group (28.6%) had more complications than the enucleation group (18.6%), but this difference was not statistically significant (*p* = 0.524). Two occurred early (within 3 months postoperatively): one contracted socket prior to delayed primary implant insertion post-enucleation for endophthalmitis and one edematous and inflamed socket that resolved with topical eyedrops post-enucleation. The other 13 sockets had late (more than 3 months postoperatively) complications. In Table 4, we herewith suggest segregating the various socket-related complications into mild, moderate, and severe, as described. Figure 4 describes the 13 sockets that had complications post-primary enucleation. Among the 5 sockets that had implant exposure/extrusion post-enucleation (without infection), a breakdown of the implant and wrapping material was as follows: two Medpor implants (20 mm and 23 mm) wrapped with Tutopatch, one Medpor implant (20 mm) wrapped with AlloDerm, one acrylic implant (18 mm) wrapped with Tutopatch, and one acrylic implant (20 mm) without wrapping. All were spherical in shape.

Of the seven primary eviscerations, one socket had socket discharge and mild inferior fornix shortening with lid disorder, and another had mild superior sulcus deformity. Both were late complications.

**Figure 5:**
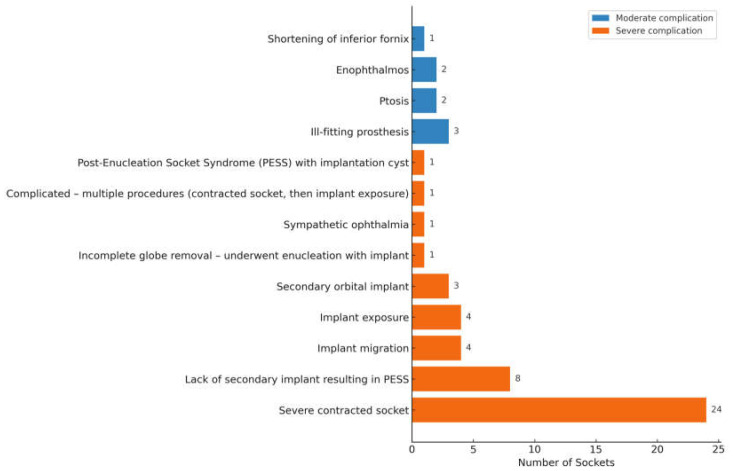
Complications in Secondarily Managed Sockets (n = 55).

Of the 88 sockets managed secondarily, 50 sockets were post-enucleation, 6 post-evisceration, 1 post-exenteration, 8 congenital anophthalmic sockets, and 23 were of unknown surgery type (ie, either enucleation or evisceration) (Table 5). Of the available data, 53 sockets had an implant present during the first review, whereas 16 lacked a definite implant. Implant status was unknown in 10. Forty-seven sockets (53.4%) had severe complications, with approximately half requiring secondary socket reconstruction. Eight sockets had moderate complications (Figure 5). Secondary interventions included fornix reconstruction, ptosis repair, customized prosthesis fitting for ill-fitting prosthesis and secondary orbital implant placement or exchange. One patient with congenital anophthalmia underwent socket reconstruction with a dermis-fat graft.

## 4. Discussion

Management of anophthalmic sockets is often complex, but the outcomes can be highly rewarding when successful. Not only should the socket be managed adequately, but there must also be consistent long-term follow-up to identify complications. Enucleation, evisceration, and exenteration surgeries, which are utilized in the setting of irreparable trauma or severe disease, may require an adjustment period postoperatively.

At our center, enucleation was the most frequently performed procedure, followed by socket reconstructions, eviscerations, and exenterations. The leading indication for globe removal was intraocular tumor, whereas in many developing countries, trauma and postoperative endophthalmitis predominate as causes of anophthalmic sockets [23,24]. Enucleation was preferred as the primary procedure in our series, as it eliminates the theoretical risk of leaving residual malignant tissue within the orbit. During the study period, enucleation was also preferred over evisceration, as it was believed that the latter disrupted the globe’s integrity and posed a theoretical risk of exposing uveal antigens, which could incite an autoimmune reaction known as sympathetic ophthalmia in the contralateral eye. However, studies have shown that the risk of sympathetic ophthalmia post-evisceration is very low [25,26,27], and a recent review by Jordan et al. highlighted sympathetic ophthalmia from all causes to be present postoperatively in both enucleation and evisceration (prevalence 0.001% and 0.002%, respectively) [28]. We also acknowledge that current consensus supports evisceration as an acceptable procedure for eyes requiring removal following trauma [29,30,31,32,33]. Although the procedure carries a minimal potential risk of sympathetic ophthalmia, this has not been definitively shown to significantly increase the risk of developing the condition [34,35,36]. Nevertheless, many of our trauma patients were migrant foreign workers who, upon repatriation to regions with limited access to tertiary care, might not receive timely management should such a complication occur, potentially resulting in irreversible consequences. In our center, we also received a higher tumor load; as such, enucleation was more likely to be performed, contributing to lower evisceration numbers. Furthermore, refinements in surgical technique over the years have enhanced both the surgeon’s confidence and preference for enucleation at our center, reinforcing its role as the primary procedure in the majority of cases.

Enucleation and evisceration are contraindicated in cases of intraocular malignancy with orbital exenteration: these patients generally require exenteration [37,38,39]. Evisceration has its benefits over enucleation. In a study by Yousuf et al., eviscerations only took about half the time needed for enucleations (47.3 ± 10.3 vs. 89.6 ± 10.1 min) [40]. Being able to remove an eye without having to disinsert and reinsert the extraocular muscles and using implants that require wrapping also carries the benefit of requiring less operative time and, therefore, less patient exposure to anesthesia. Other benefits of evisceration include being a less technically demanding alternative to enucleation, which offers improved cosmetic results, enhanced implant motility, better overall patient outcomes, and reduced psychological distress, since part of the eye is preserved rather than being completely removed [30,31,41,42,43]. It is noted that among surveyed ocularists, a majority (92%) chose evisceration as their primary choice for patients requiring eye removal [43].

In our series, the majority of patients had implant wrapping, with donor sclera (52.5%) being the most commonly used material due to its ready availability from the local eye bank and its ease of use. Wrapping orbital implants not only acts as a protective barrier against exposure, but also aids in muscle support suturing, thereby retaining the implant within the orbit and also imparting implant motility, which leads to better ocular prosthetic motility [44,45]. Other proposed wrapping materials include, but are not limited to, porcine collagen, fascia lata, human rectus abdominal sheath, and posterior auricular muscle [46]. Not all surgeons, however, routinely wrap implants, as extraocular muscles can be sutured directly to porous polyethylene spheres [46]. Importantly, wrapping does not guarantee prevention of exposure. In our cohort, we found that implant exposure was the most common complication following enucleation, consistent with previous reports [47,48]. At our center, Medpor was the most commonly used implant, followed by acrylic. This is due to the theoretical idea that porous implants have the advantage of promoting fibrovascular ingrowth that allows them to integrate into the orbital tissue, which potentially decreases the chance of extrusion and improves motility [3,49,50,51]. However, there has yet to be conclusive data to support the use of porous over non-porous implants [52]. In fact, several studies have failed to demonstrate any motility advantages of porous implants over nonporous implants [53,54,55]. Furthermore, implant exposure was the most common and challenging problem associated with porous orbital implants [56]. Reported exposure rates for nonporous spherical implants were generally low (0–3%) [52,56,57,58,59]. Exposure rates for porous orbital implants were generally low as well; however, they may vary from 0% to 50% [52,56,57,60,61,62]. Wladis et al. similarly found that exposure and extrusion rates were comparable between porous and nonporous material implants [52], suggesting that implant type might not lead to a significant difference in this complication. Therefore, limited evidence suggests that porous implants do not significantly lower extrusion or exposure rates, and in fact, may increase them [56,63,64].

The total complication rate of 18.5% obtained in our study post-primary procedure (enucleation and evisceration) is comparable to previous reports, which range from 16% to 34% for enucleations and 11% to 20% for eviscerations [40,47,48,57,65]. However, a study by Nguyen et al. showed that their enucleation surgeries (35.7%) had more postoperative complications than the evisceration group (15.6%) (*p* = 0.004), which is the opposite for our cohort: evisceration group (28.6%) versus enucleation group (18.6%). This could be attributed to our low evisceration numbers (seven) and hence limited statistical power to detect true differences between groups. In addition, all our eviscerations were performed for fulminant endophthalmitis, a setting in which postoperative inflammation, socket edema, and scarring may predispose patients to higher complication rates, unlike eviscerations performed for non-infective causes in other studies.

A survey among oculofacial surgeons in the Asia–Pacific region reported silicone and acrylic as the most frequently used orbital implant materials, with porous polyethylene ranking second for both adult and pediatric patients [66]. In our series, only one socket received a hydroxyapatite implant, which subsequently developed enophthalmos 2 years postoperatively. This was corrected with the insertion of Medpor wedges.

Following enucleation or evisceration, restoration of orbital volume is essential. The implant provides structural support for the ocular prosthesis and enhances postoperative cosmesis, while possessing good biocompatibility, supporting anophthalmic orbit growth and minimal complication rates [3,64,67,68]. Culler emphasized the need for complete orbital volume replacement during enucleation, noting that restoring the exact amount of tissue removed should be the central goal of reconstruction [69]. Kaltreider et al. and Custer et al. demonstrated considerable variability in axial length and orbital volume, ranging from 6.9 mL to 9.0 mL [70,71]. These findings underscore the importance of tailoring implant size to each patient to achieve accurate volume replacement and optimal cosmetic outcomes. Intraoperatively, the surgeon uses sizer spheres and determines whether the soft tissues can cover the anterior face of the implant without tension. A cold test tube is placed within the orbit before sizing to prevent under-sizing of the implant from soft tissue edema.

Myoconjunctival enucleation was the preferred technique employed by the surgeon in this study. Key differences between myoconjunctival enucleation and conventional enucleation include the diagonal closure of the anterior Tenon’s capsule, followed by the passage of the four recti muscle sutures through the overlying conjunctiva. Specifically, the superior and inferior recti muscles are anchored 12 mm from the horizontal edge of the conjunctival incision within their respective fornices. In contrast, the medial and lateral rectus muscles are attached myoconjunctivally to the medial and lateral fornices, positioned 25 mm apart [72]. By suturing the extraocular muscles to the fornices and the implant in their normal anatomic positions, implant stability and prosthetic motility are increased, resulting in better outcomes [73].

Iris-painted conformers are preferred in our center as they provide immediate postoperative cosmetic benefits and aid in rehabilitating not just the patient but also the family member(s), thereby improving psychosocial well-being. Anecdotally, patients often comment that it is the actual iris color that is the most important consideration in a permanent prosthetic eye [74]. Moreover, custom-made conformers can also be used to expand small or contracted sockets, stimulate eyelid movement, aid hygiene, guide the clinician in shaping the definitive prosthesis and reduce the need for post-insertion adjustments.

At our center, customized prostheses have evolved over the years, gaining greater acceptance. We have an in-house ocularist who oversees all our prosthesis creation. This allows tailoring of the prosthesis to the patient. Where possible, our center also favors insertion of customized permanent prosthesis, bearing in mind economic factors. A custom-made ocular prosthesis provides a more precise and satisfactory aesthetic appearance, particularly for those who have lost ocular structures through orbital evisceration or orbital enucleation [75].

Orbital exenteration is both psychologically and anatomically disfiguring and is rarely performed in our center. It is reserved for patients with potentially life-threatening malignancies or relentlessly progressive conditions that are unresponsive to other treatments. All the cases conducted in our center were performed for malignant disease. Of the five patients who had exenteration, three passed on within the year due to metastatic spread, which limited the availability of long-term data on postoperative complications.

The two most common complications in secondarily managed sockets were severely contracted sockets (n = 24) and post-enucleation socket syndrome from the absence of orbital implant (n = 8). More than half of the contracted sockets were attributable to undersized orbital implants, underscoring the importance of selecting an implant that provides sufficient primary volume replacement. When acceptable to the patient, socket reconstruction with implant exchange was the preferred management strategy. Common approaches involved tissue grafting (with buccal mucous membrane or dermis fat graft) to increase the surface area, fornix-forming sutures to deepen the fornix and dermis fat graft or implant exchange to correct volume loss.

Dermis fat grafts provide a cost-effective alternative to alloplastic orbital implants, while eliminating risks of extrusion and foreign body reaction [76]. Beyond volume replacement, they serve as a biologic scaffold, supporting conjunctival suturing and advancement and enhancing ocular surface reconstruction. The intrinsic vascularization of the graft enhances tissue integration and reduces the likelihood of fat atrophy over time. Preservation of conjunctival fornices can be achieved by suturing the conjunctival remnants directly to the graft margins, while graft contouring allows for the recreation of deep fornices and improved prosthetic motility and overall cosmetic rehabilitation [77]. In some contracted sockets, the addition of a customized prosthesis alone improved the aesthetic outcome to the patient’s satisfaction, removing the need for further surgical intervention.

## 5. Conclusions

At our center, ocular tumors and trauma were the most common indications leading to anophthalmic sockets. Given that a substantial proportion of patients developed postoperative complications, ophthalmologists must remain vigilant and up-to-date with the evolving approaches to socket management to optimize both functional and cosmetic outcomes.

## Figures and Tables

**Table 1:** Predisposing Conditions for Primary and Secondary Anophthalmic Socket Management.

	All Procedures (n = 170)		Enucleation (n = 120)		Evisceration (n = 13)	
Condition	Primary Procedure (n = 82) (%)	Secondary Socket Management (n = 88) (%)	Primary Procedure (n = 70) (%)	Secondary Socket Management (n = 50) (%)	Primary Procedure (n = 7) (%)	Secondary Socket Management (n = 6) (%)
Ocular/Orbital Tumor	41 (50.0)	31 (35.2)	36 (51.4)	25 (50.0)	0 (0.0)	2 (33.3)
Trauma	14 (17.1)	31 (35.2)	14 (20.0)	15 (30.0)	0 (0.0)	2 (33.3)
Fulminating infections	12 (14.6)	8 (9.1)	5 (7.14)	1 (2.0)	7 (100.0)	1 (16.7)
Congenital anomalies	5 (6.1)	9 (10.2)	5 (7.14)	2 (4.0)	0 (0.0)	0 (0.0)
Rubeotic glaucoma	6 (7.3)	2 (2.3)	6 (8.57)	1 (2.0)	0 (0.0)	1 (16.7)
Failed retinal detachment surgery	4 (4.9)	1 (1.1)	4 (5.71)	0 (0.0)	0 (0.0)	0 (0.0)
Others	0 (0.0)	10 (11.4)	0 (0.0)	6 (12.0)	0 (0.0)	0 (0.0)

**Table 2:** Breakdown of Primary Sockets (n = 82).

Demographics	n (%)
Gender	
Male	45 (54.9)
Female	37 (45.1)
Nationality	
Singaporean	44 (53.7)
Indonesian	15 (18.3)
Vietnamese	7 (8.5)
Indian	5 (6.1)
Malaysian	5 (6.1)
Others (East Timorese	6 (7.3)
Bruneian, Burmese, Malaysian)	
Mean Age	34.1 ± 30.0 years
Enucleation Evisceration Exenteration	70 (85.4) 7 (8.5) 5 (6.1)
Mean follow-up period	3.1 ± 3.4 years

**Table 3:** Shapes and Materials Used for Implants.

Implant Characteristics	n (%)
Implant shapes for sockets that underwent primary procedure (n = 75)	
Spherical	72 (96.0)
Conical	2 (2.67)
Quadraspheric	1 (1.33)
Materials used for the wrapped implants (n = 59)	
Donor sclera	31 (52.5)
Bovine pericardium (Tutopatch^®^ (LifeCell Corporation, New Jersey, USA))	27 (45.8)
Acellular dermal matrix (AlloDerm^TM^ (Tutogen Medical GmbH, Neunkirchen am Brand, Germany))	1 (1.69)

**Table 4:** Examples of Postoperative Complications.

Complications	Description
Mild	Discomfort, pain, inflammation, edema etc.
Moderate	Conjunctival prolapse, wound dehiscence, delayed recovery, ptosis, mild enophthalmos, etc.
Severe	Incomplete globe removal, implant extrusion, exposure, severe contracted sockets, severe infections, revision surgery, etc.

**Table 5:** Breakdown of Secondarily Managed Sockets (n = 88).

Demographics	n (%)
Gender	
Male	42 (47.7)
Female	46 (52.3)
Nationality	
Singaporean	49 (55.7)
Indonesian	14 (15.9)
Others (Philippines, Russian, Burmese, Sri Lankan, Cebu, Chinese, German)	7 (8.0)
Malaysian	
Bangladeshi	6 (6.8)
Vietnamese	3 (3.4)
Bruneian	3 (3.4)
Indian	3 (3.4)
Mean Age	38.3 ± 24.7 years
Primary Procedure Performed	
Enucleation	50 (56.8)
Unknown	23 (26.1)
Evisceration	6 (6.8)
Congenital anophthalmia	8 (9.1)
Exenteration	1 (1.1)

## Data Availability

Data will be made available upon request.
